# ZCCHC10 suppresses lung cancer progression and cisplatin resistance by attenuating MDM2-mediated p53 ubiquitination and degradation

**DOI:** 10.1038/s41419-019-1635-9

**Published:** 2019-05-28

**Authors:** Yichong Ning, Na Hui, Bei Qing, Yiming Zhuo, Wei Sun, Yan Du, Shunlian Liu, Kaili Liu, Jianlin Zhou

**Affiliations:** 1 State Key Laboratory of Developmental Biology of Freshwater Fish, College of Life Science, Hunan Normal University, 36 Lushan Road, Changsha, Hunan China; 2 Key Laboratory of Protein Chemistry and Developmental Biology of the Ministry of Education, College of Life Science, Hunan Normal University, 36 Lushan Road, Changsha, Hunan China; 3 Department of Thoracic Surgery, The Second Xiangya Hospital, Central South University, 139 Renmin Road, Changsha, Hunan China

**Keywords:** Tumour-suppressor proteins, Ubiquitylation

## Abstract

The activation of p53 tumor suppressor is essential for preventing abnormal cell proliferation and carcinogenesis. ZCCHC10 was previously identified as a potential p53-interacting partner in a yeast two-hybrid screen, but the interaction in cells and its subsequent influence on p53 activity and cancer development have not been investigated. In this paper, we demonstrate that ZCCHC10 expression levels are statistically lower in lung adenocarcinoma tissues than the corresponding adjacent noncancerous tissues, and decreased expression of ZCCHC10 mRNA predicts poorer survival of the patients. Ectopic expression of ZCCHC10 in lung cancer cells harboring wild-type p53 dramatically suppresses cell proliferation, colony formation, migration, invasion and cisplatin resistance in vitro, as well as tumor growth and metastasis in vivo. Conversely, knockdown of ZCCHC10 exerts opposite effects in the normal lung cell Beas-2b. However, ZCCHC10 has no influence on the biological behaviors of p53-null (H358) or p53-mutant (H1437) lung cancer cells. Mechanistically, ZCCHC10 binds and stabilizes p53 by disrupting the interaction between p53 and MDM2. The p53 inhibitor pifithrin-α attenuated the influences of ZCCHC10 overexpression on p53 pathway, cell cycle, apoptosis, and epithelial-mesenchymal transition, whereas the p53 activator Nutlin3 could reverse the effects of ZCCHC10 knockdown. Collectively, our results indicate that ZCCHC10 exerts its tumor-suppressive effects by stabilizing the p53 protein and can be used a potential prognostic marker and therapeutic target in lung adenocarcinoma.

## Introduction

Lung cancer is the leading cause of cancer-related death among males and the second leading cause of cancer-related death (after breast cancer) among females^1^. Lung cancer is histologically classified into small cell lung cancer (SCLC) and non-small cell lung cancer (NSCLC). NSCLC accounts for most (~85%) of lung cancer cases and includes three subtypes: adenocarcinoma (LUAD), squamous cell carcinoma (LUSC), and large cell carcinoma (LCC). LUAD and LUSC are the two most predominant subtypes of NSCLC^2^.

A common feature of all human cancers is the loss of p53 function, either via mutation or inactivation^3,4^. Mutations in the TP53 gene, which encodes the p53 protein, occur in approximately half of all cancer specimens^5^. In the remaining cancers that retain wild-type p53 (wtp53), p53 function is frequently abolished by the overexpression of p53 inhibitors, such as MDM2^6^ and QCR2^7^, or the silencing of p53 activators, such as BAI1^8^ and LACTB^9^. The tumor suppressor p53 mainly functions as a transcription factor, whose subcellular localization, protein stability, DNA-binding property, and transactivation activity are regulated by covalent modifications and/or physical interactions with other proteins^3,4^. Among these proteins, the E3 ubiquitin ligase MDM2 plays a central role in governing p53 levels and activity, as it directly ubiquitinates and targets the p53 protein for proteasomal degradation^6^ or nuclear export^10^. Many proteins have been found to regulate p53 stability by affecting the interaction between p53 and MDM2. For example, LACTB stabilizes the p53 protein by disrupting the p53-MDM2 interaction^9^; HMGA2 directly interacts with both p53 and MDM2 to enhance MDM2-mediated p53 ubiquitination and degradation^11^.

ZCCHC10 (zinc finger CCHC-type containing 10) gene is located on chromosome 5q31.1, a region that is frequently deleted or silenced by hypermethylation in lung cancer^12^, gastric cancer^13^, and acute myeloid leukemia^14^. Genome-wide association study indicated the potential role of the 5q31.1 region in both lung cancer development and the effects of cigarette smoking^15^. Several tumor suppressor genes have been identified in this region, including IRF1, RAD50 and GDF9^16^. Thus, ZCCHC10 is possibly a candidate tumor suppressor. Stelzl et al.^17^ identified ZCCHC10 to be a potential p53-interacting partner by a yeast two-hybrid method, but the interaction in cells and its subsequent influence on p53 activity and cancer development have not been investigated.

In this study, we demonstrated that ZCCHC10 protein interacts with and stabilizes p53 protein by interfering MDM2-mediated ubiquitination of p53. ZCCHC10 is downregulated in LUAD tissues and ectopic expression of ZCCHC10 inhibits lung cancer progression and cisplatin resistance in a p53-dependent manner.

## Materials and methods

### Tissue specimens

Fifty-four pairs of fresh lung cancer and adjacent noncancerous tissues were collected from the Second Xiangya Hospital, Central South University. The clinicopathological characteristics are listed in Supplementary Table S1. The samples were histologically confirmed by HE (hematoxylin and eosin) staining. This study was approved by the Ethics Committee of the Second Xiangya Hospital.

### Plasmids, antibodies, and chemicals

The ZCCHC10 coding sequence (NM_017665.3) was cloned into the pCMV-Myc and pEGFP-C3 vectors (Clontech, Mountain View, CA, USA), generating the Myc-ZCCHC10 (Myc-Zh10) and EGFP-ZCCHC10 plasmids. The Myc-Zh10 was used a template of PCR to construct a Myc-mtZh10 plasmid, in which the first two Cys residues of the CCHC-type zinc finger (C^45^QKCLEFGHWTYEC^58^) were substituted with Ala. The Myc-MDM2 plasmid was constructed by inserting the MDM2 coding sequence into the pCMV-Myc vector, and used a template of PCR to construct Myc-MDM2C464A plasmid, in which the Cys at residue 464 was mutated into Ala. HA-p53 plasmid was described previously^18^. Antibodies and chemicals used in this study are listed in Supplementary Table S2.

### Survival analysis

Kaplan–Meier survival curves with hazard ratio (HR), 95% confidence interval (CI) and log rank *p*-value (*p*) were calculated and plotted using the Kaplan–Meier plotter (http://kmplot.com) platform^19^, which has integrated the gene expression data, relapse-free and overall survival information from GEO (Gene Expression Omnibus), EGA (European Genome-phenome Archive) and TCGA (The Cancer Genome Atlas).

### Cell culture, transfection, and luciferase assay

Hek293, HeLa, Beas-2b, A549, H460, MCF7, HBL100, HCT116, H358, and H1437 cells were obtained from ATCC (Manassas, VA, USA) and cultured according to the instruction of ATCC. A549-luc cell line was purchased from Hanbio Biotechnology Co., Ltd (Shanghai, China). All the cell lines were mycoplasma-free and authenticated using short tandem repeats analysis by Yubo Biological Technology Co., Ltd. (Shanghai, China). Transfection and luciferase assay were performed as described previously^18^.

### Lentivirus infection

The lentiviruses over-expressing ZCCHC10 gene and empty vector were purchased from GeneChem (Shanghai, China), lentiviruses expressing ZCCHC10 shRNA and scramble shRNA (CSHCTR001-LVRH1MP) were obtained from GeneCopoeia (Guangzhou, China). The target sequences of ZCCHC10 shRNAs are listed in Supplementary Table S3. Infection and selection for stable cell lines were performed as described previously^20^.

### Immunofluorescence staining

Beas-2b cells were seeded on glass coverslips, fixed, and sequentially incubated with the primary antibody and fluorescently labeled secondary antibody, as described previously^18^.

### Co-immunoprecipitation (co-IP)

Co-IP experiments were performed using endogenous proteins in Beas-2b and overexpressed proteins in Hek293 cells as previously described^18,20^. For analyzing the effect of ZCCHC10 on the interaction between p53 and MDM2, Hek293 cells were co-transfected with 3 μg of HA-p53 plasmid, 3 μg of Myc-MDM2C464A plasmid and an increasing amount (0, 3, 6, 9 μg) of EGFP-ZCCHC10 plasmid. At 24 h post transfection, co-IP assay was performed.

### Ubiquitination assay

The cell lysates were extracted and immunoprecipitated with an anti-p53 antibody after cells were treated with MG132 for 4 h. The precipitated proteins were subjected to western blotting (WB) using an anti-ubiquitin antibody to detect the ubiquitinated p53 protein.

### MTT assay, colony formation assay, wound healing, and transwell invasion assays

MTT assay, colony formation assay, wound healing assay, and transwell invasion assay were performed as described previously^20^.

### Cell cycle and apoptosis analysis

Cell cycle and apoptosis was analyzed by PI (propidium iodide) staining kit (Muti Sciences, Hangzhou, China) and Annexin-V-FITC/PI staining kit (Invitrogen), respectively, according to the manufacturer’s instructions. After staining, cells analyzed using a flow cytometer (CytoFlex, Beckman Coulter, Fullerton, CA, USA).

### Xenograft tumor formation and metastasis assay

BALB/c nude male mice (4–6-weeks-old) were purchased from SLACCAS Jingda (Changsha, China). For generating a xenograft tumor model, a total of 1 × 10^7^ the A549 cells stably over-expressing ZCCHC10 and empty vector into the left and right dorsal flanks of nude mice, respectively. The tumor volume was measured as described previously^20^. At 56 days after injection, mice were sacrificed and the tumors were surgically dissected, embedded in paraffin for HE and IHC staining. For tumor metastasis assay, the A549-luc cells stably over-expressing ZCCHC10 or empty vector were implanted into nude mice by tail-vein injection. Tumor metastasis was monitored biweekly by IVIS Lumina XR in vivo imaging system (Caliper Life Sciences, Mountain View, CA, USA). At 62 days after injection, mice were sacrificed, the tumor nodules formed on the lung and liver surfaces were counted, lungs and livers were embedded in paraffin for HE staining.

### Statistical analysis

All statistical analyses were conducted with SPSS 22.0 (SPSS Inc., Chicago, IL). The values are presented as means ± SD. Differences between two groups were analyzed using Student’s *t*-test, and the frequencies of two groups were compared using the chi-squared test.

## Results

### ZCCHC10 is downregulated in LUAD and decreased ZCCHC10 expression predicts poor prognosis

**Fig. 1:**
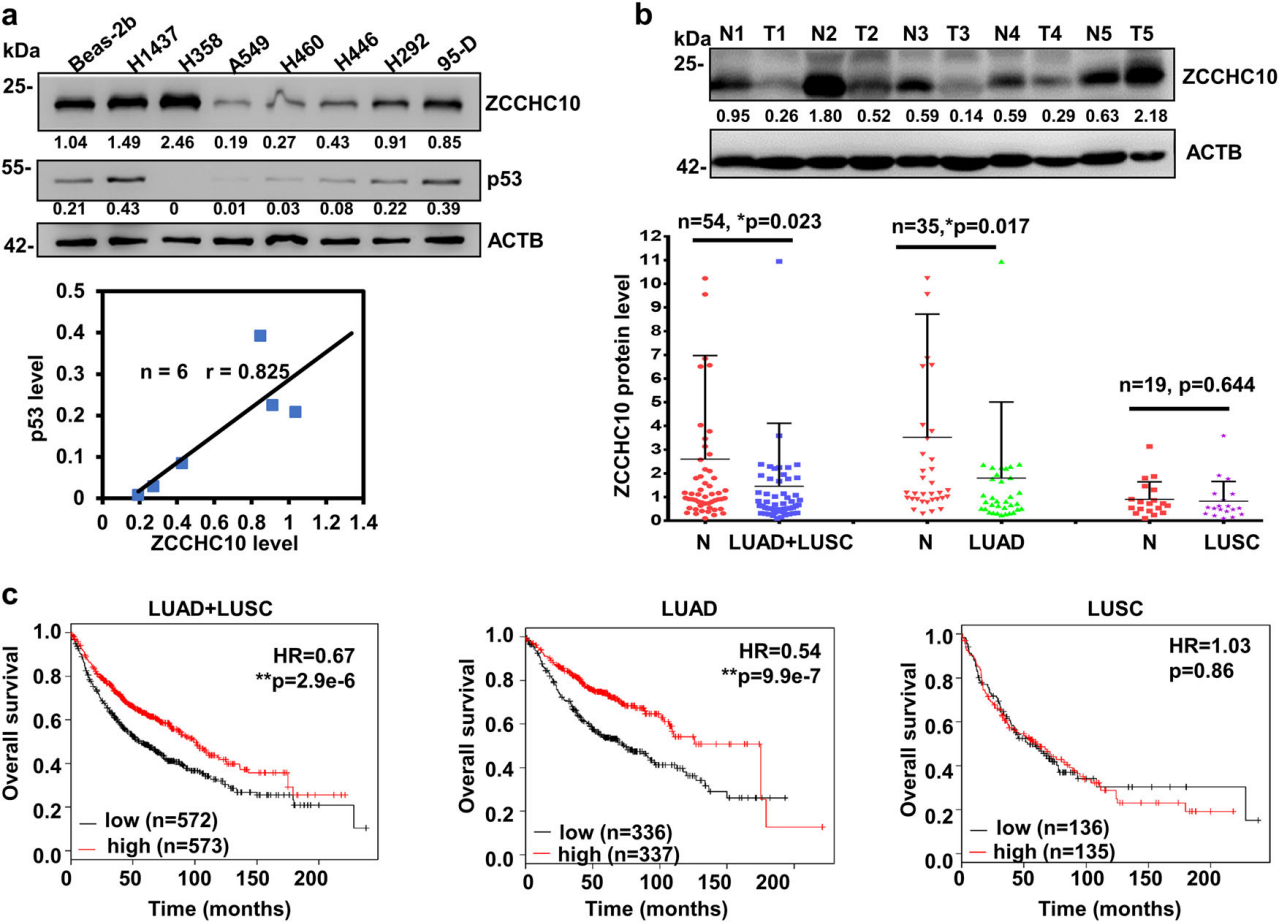
Expression of ZCCHC10 protein and prognostic role of ZCCHC10 mRNA expression in lung cancer. **a** Western blotting (WB) analysis of ZCCHC10 and p53 proteins in lung cell lines. The numbers below the blots indicate the relative band intensity of ZCCHC10 protein and p53 protein normalized against that of ACTB. Pearson’s correlation coefficient (*r*) between p53 and ZCCHC10 in the six lung cell lines with wtp53 was assessed based on the band intensity. **b** WB analysis of ZCCHC10 in 54 pairs of lung cancer (T) and the matched noncancerous (N) tissues. A representative image and statistical plots were shown in upper and lower panel, respectively. Means ± SD shown are representative of two independent experiments and analyzed by two-tailed student *t*-test, *p ≤ 0.05. **c** Effect of ZCCHC10 mRNA expression in lung cancer tissue on patient overall survival was assessed based on the database of Kaplan–Meier plotter platform

Through analyzing cancer microarray database on the Oncomine platform, we found that the levels of ZCCHC10 mRNA in LCC, LUAD, and LUSC tissues were significantly lower than lung normal tissues (foldchange: −1.520, −1.334, and −1.19, respectively) (Supplementary Fig. S1). Then, we detected ZCCHC10 protein levels in lung cell lines and tissues. Among the eight lung cell lines detected in this study, ZCCHC10 protein levels in H358 (p53-null) and H1437 (p53-mutant) cells were the highest (Fig. 1a). However, in other six cell lines (Beas-2b, A549, H460, H446, H292, and 95-D), which harbor wild-type p53 (wtp53), the normal lung epithelial cell Beas-2b had the highest level of ZCCHC10 protein (Fig. 1a). Interestingly, p53 protein levels positively correlated with ZCCHC10 protein levels in the cell lines with wtp53 (Fig. 1a). In 54 pairs of primary lung cancer and corresponding adjacent noncancerous tissues, the ZCCHC10 protein was expressed at significantly lower levels in the lung cancer (including LUAD and LUSC) and LUAD tissues than in the normal tissues, but the difference in ZCCHC10 expression between LUSC and normal tissues was not significant (Fig. 1b). ZCCHC10 expression was significantly associated with cancer subtypes (*p* = 0.0071), but was not associated with gender, age, differentiation, lymph node metastasis or tumor size (Supplementary Table S4).

Kaplan–Meier Plotter analysis showed that the level of ZCCHC10 mRNA expression was positively correlated with overall survival in all lung cancer patients (HR = 0.67, CI = 0.57–0.80, *p* = 2.9e-6) and LUAD patients (HR = 0.54, CI = 0.42–0.70, *p* = 9.9e-7), but not in LUSC patients (HR = 1.03, CI = 0.75–1.4, *p* = 0.86) (Fig. 1c). Moreover, the lung cancer patients who never smoke (HR = 0.26, CI = 0.009–0.69, *p* = 0.0035) benefited more from ZCCHC10 expression than the patients who smoke (HR = 0.56, CI = 0.36–0.85, *p* = 0.0066). (Supplementary Fig. S2). Based on these results, ZCCHC10 expression is a prognostic marker for lung cancer, particularly LUAD.

### ZCCHC10 inhibits the proliferation, migration, and invasion of the lung cancer cells with wtp53

**Fig. 2:**
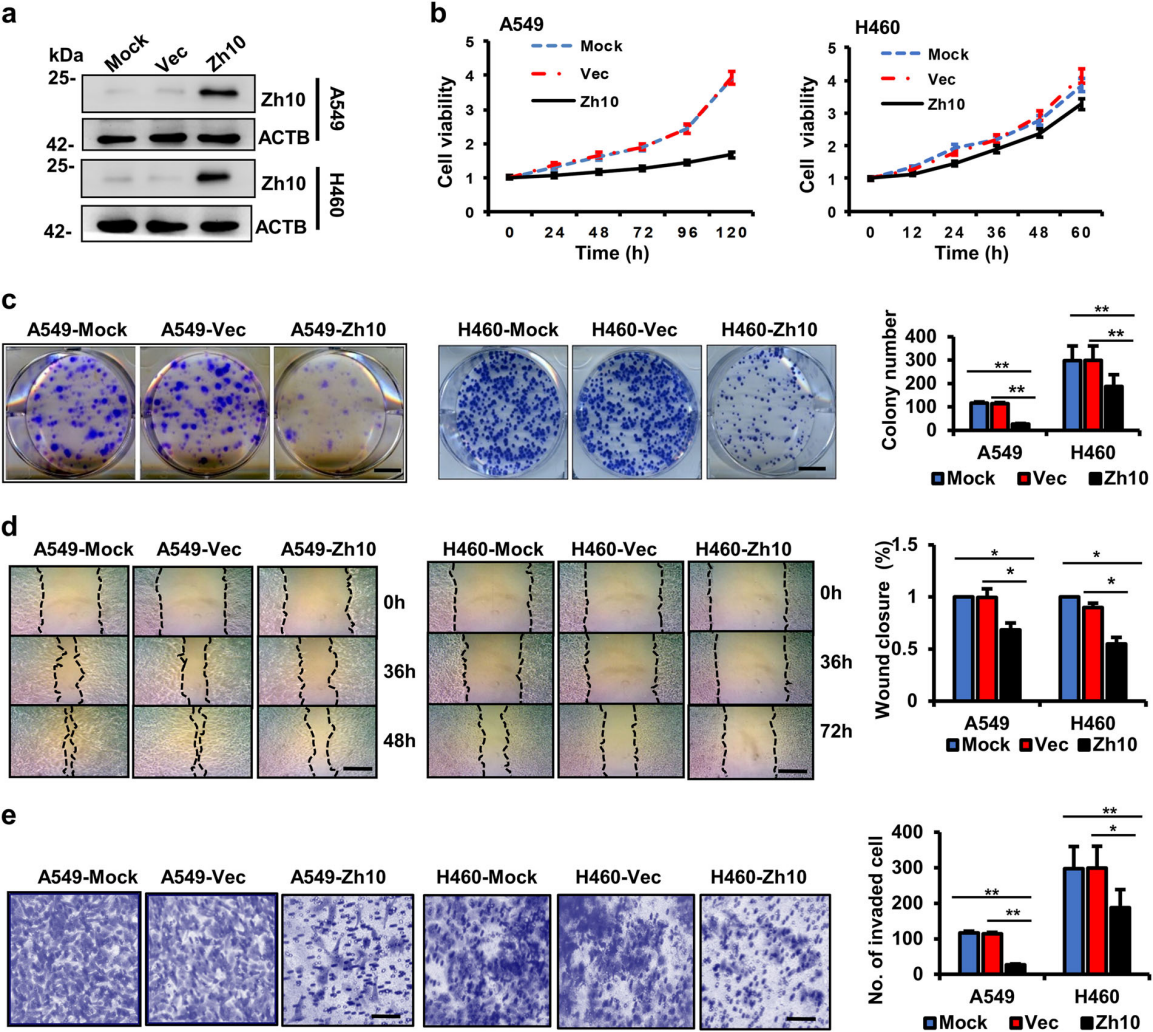
Ectopic expression of ZCCHC10 inhibits proliferation, migration, and invasion of lung cancer cells A549 and H460. **a** WB analysis of ZCCHC protein in the untreated cells (Mock) and the cells with stably over-expressing ZCCHC10 (Zh10) or empty vector (Vec). **b** The cell viability was determined by MTT assay. **c** Representative images of colony (left panel) and the colony numbers (right panel). Bar length: 1 cm. **d** Representative images (left panel) of wound area at the indicated time and percentage of wound closure at 48 h (A549) or 72 h (H460) after scratching (right panel). Bar length: 50 μm. **e** Representative images (left panel) and statistical analysis (right panel) of invaded cells in the Transwell invasion assay. Bar length: 50 μm. All the values were presented as means ± SD for three independent experiments. Differences between two groups were analyzed by student *t*-test; *p ≤ 0.05 and **p ≤ 0.01

To explore the roles of ZCCHC10 in lung cancer, we used the lentivirus-mediated expression system to stably over-express ZCCHC10 in A549 and H460 cell lines, which lack endogenous ZCCHC10 (Fig. 2a). According to the results of the MTT assay, cell proliferation was inhibited by ZCCHC10 overexpression (Fig. 2b). In the colony formation assay, a much fewer number of colonies was observed in cells over-expressing ZCCHC10 compared with mock-infected cells or cells infected with the empty vector lentivirus (Fig. 2c). Wound healing assay showed that cells over-expressing ZCCHC10 closed the scratched wound at a slower rate than the control cells (Fig. 2d). Transwell invasion assay observed that compared to the control cells, significantly fewer cells stably over-expressing ZCCHC10 had migrated (Fig. 2e). These results indicated that ZCCHC10 inhibits the proliferation, colony formation, migration, and invasion of lung cancer cells.

**Fig. 3:**
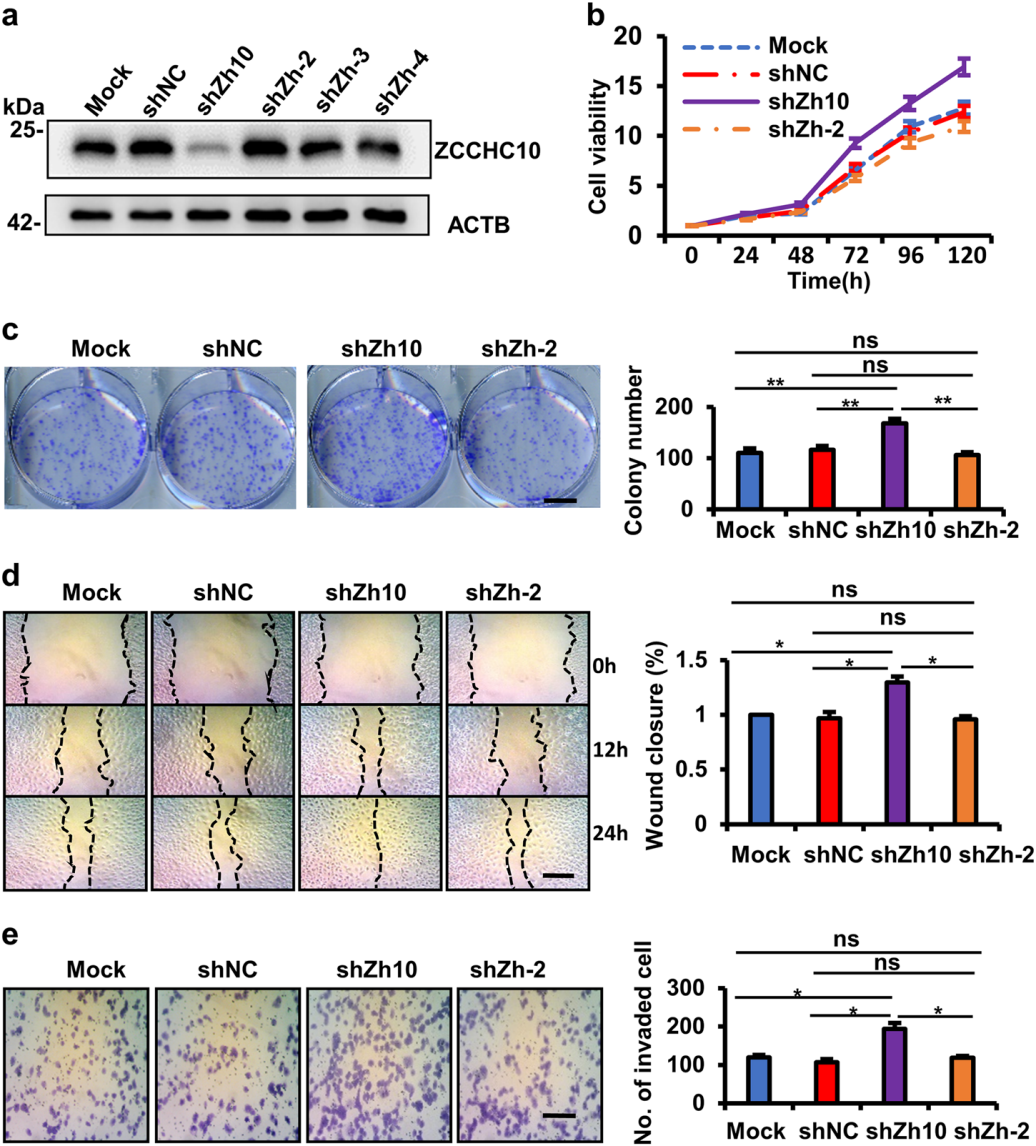
ZCCHC10 knockdown promotes proliferation, migration, and invasion of the normal lung epithelial cell line Beas-2b. **a** ZCCHC protein levels in the untreated cells (Mock) and the cells with stably expressing ZCCHC10 shRNAs (shZh10, shZh-2, shZh-3, and shZh-4) or scramble shRNA (shNC). **b** The cell viability was determined by MTT assay. **c** Representative images of colony (left panel) and the colony numbers (right panel). Bar length: 1 cm. **d** Representative images (left panel) of wound area at the indicated time and percentage of wound closure at 24 h after scratching (right panel). Bar length: 50 μm. **e** Representative images (left panel) and statistical analysis (right panel) of invaded cells in the Transwell invasion assay. Bar length: 50 μm. All the values were presented as means ± SD for three independent experiments. Differences between two groups were analyzed by student *t*-test; *p ≤ 0.05 and **p ≤ 0.01

To further confirm the anti-tumor effects of ZCCHC10, the ZCCHC10 gene was silenced by lentivirus-delivered shRNAs in the Beas-2b cells, which harbors high level of endogenous ZCCHC10. We designed four lentivirus-delivered shRNAs that target different regions of the ZCCHC10 mRNA, and observed that only the shZh10 could reduce effectively the level of ZCCHC10 protein (Fig. 3a). In vitro experiments demonstrated that, compared to mock or scramble shRNA transduction, shZh10 transduction in Beas-2b cells increased cell proliferation, colony formation, migration, and invasion; but the ineffective shZh-2 had no significant effects (Fig. 3b–e). Likewise, ZCCHC10 knockdown also promoted cell proliferation and migration in Hek293 cells (Supplementary Fig. S3). However, overexpression or knockdown of ZCCHC10 gene had no significant effects on the proliferation, colony formation, migration, or invasion of p53-null (H358) or p53-mutant (H1437) cells (Supplementary Figs. S4 and S5). It suggests that ZCCHC10 exerts anti-oncogenic function in a p53-dependent way.

### ZCCHC10 inhibits tumor growth and metastasis in xenograft mouse models

**Fig. 4:**
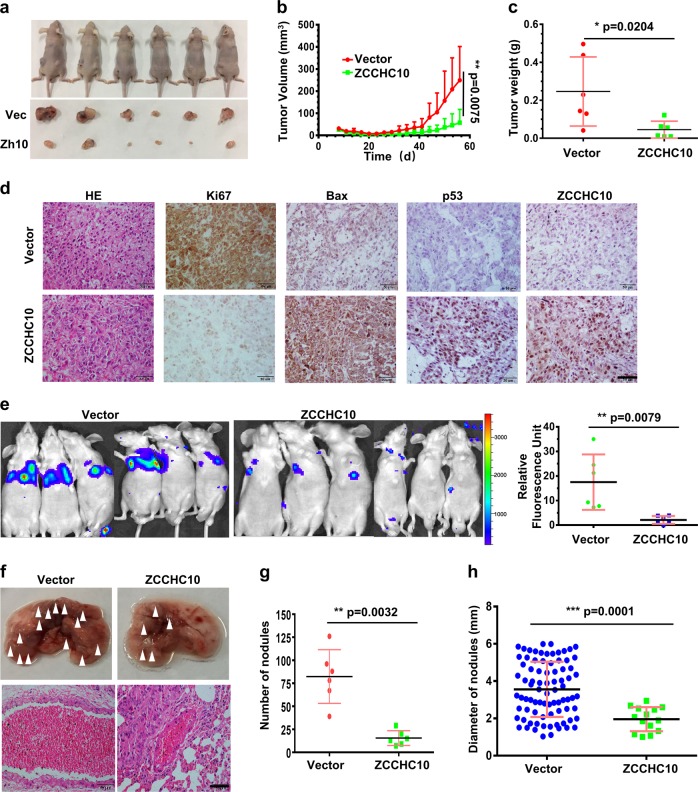
Ectopic expression of ZCCHC10 inhibits tumor growth and metastasis in xenograft mouse models. **a**–**d** ZCCHC10 overexpression in A549 cells inhibits the growth of xenograft tumors. About 1 × 10^7^ of A549 cells stably expressing empty vector or ZCCHC10 into the left and right dorsal flanks of nude mice (*n* = 6), respectively. **a** Images of the nude mice and their xenograft tumors at 56 days after injection. **b** Dynamic volume of xenograft tumors at different time after injection. **c** Weight of xenograft tumors at 56 days after injection. **d** HE and IHC staining of xenograft tumors. **e**–**h** ZCCHC10 overexpression in A549-luc cells inhibits tumor metastasis. About 5 × 10^6^ of the A549-Luc cells stably expressing ZCCHC10 or empty vector were implanted into nude mice (*n* = 6 per group) by tail-vein injection. **e** In vivo bioluminescence signals at 62 days after injection. **f** Pictures of lung (upper panel) and HE staining of lung tissue (lower panel), the arrows indicate the metastatic nodules on the surface of lungs. Bar length: 50 μm. The number (**g**) and the diameter (**h**) of nodules on lungs were quantified at 62 days after tail-vein injection

To confirm the tumor-suppressive function of ZCCHC10 in vivo, we generated subcutaneous xenograft models. The results showed that the growth rate and average weight of the tumors derived from A549-ZCCHC10 cells were much less than controls (Fig. 4a–c). HE staining showed that cells were more loosely arranged in the tumors derived from A549-ZCCHC10 cells (Fig. 4d). IHC analysis further confirmed that ZCCHC10 overexpression inhibited the expression of the proliferation marker Ki67 (Fig. 4d). Moreover, tumors derived from A549-ZCCHC10 cells had higher levels of p53 and Bax proteins than control tumors (Fig. 4d). These results suggested that ZCCHC10 inhibited tumor growth possibly through p53 pathway.

To investigate the effects of ZCCHC10 on metastasis, the A549-Luc cells stably over-expressing ZCCHC10 or empty vector (designated as A549-luc-Zh10 and A549-luc-Vec, respectively) were implanted into nude mice by tail-vein injection. In vivo bioluminescence imaging showed the control A549-luc-Vec cells produced lung metastases, whereas the animals implanted with A549-luc-Zh10 cells showed minimal or no signal (Fig. 4e). Macroscopic metastatic nodules in lungs were counted, and metastatic lesions in lungs were detected by HE staining (Fig. 4f). In consistent with the result of in vivo bioluminescence imaging, there were fewer and smaller metastatic nodules in lungs of the animals implanted with A549-luc-Zh10 than the control group (Fig. 4f–h). These results indicated that ectopic expression of ZCCHC10-suppressed metastasis of lung cancer cells.

### ZCCHC10 sensitizes lung cancer cells to cisplatin treatment by promoting p53 induction

**Fig. 5:**
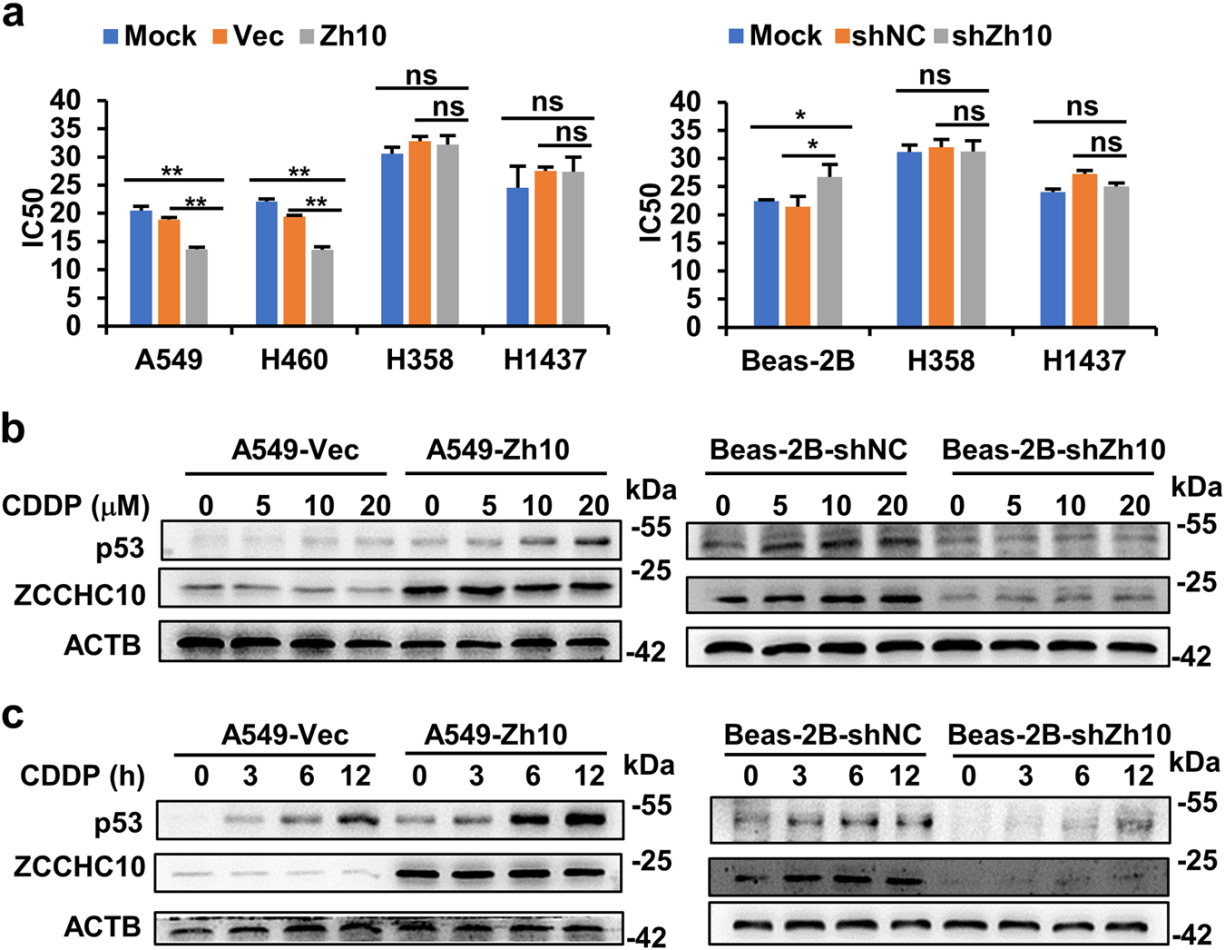
ZCCHC10 enhances cisplatin sensitivity of lung cancer cells with wtp53 through promoting p53 induction. **a** IC50 of cisplatin following overexpression or knockdown of ZCCHC10. The cells were treated with different concentration of cisplatin for 48 h. Cell viability was detected by MTT assay. **b**, **c** WB analysis of p53 protein after treatment with different concentration of cisplatin for 12 h (**b**), or with 20 μM of cisplatin for different times (**c**). *p ≤ 0.05; **p ≤ 0.01; ns: non-significance

To examine the effects of ZCCHC10 on the cisplatin sensitivity, IC50 (half inhibitory concentration) values were calculated based on the cell viability after exposure in different concentrations of cisplatin for 48 h. Based on the IC50 values, ZCCHC10 overexpression enhanced the cisplatin sensitivity in A549 and H460 cells, whereas knockdown of ZCCHC10 inhibited cisplatin sensitivity in Beas-2b cells (Fig. 5a). The same results were obtained in HeLa cells (Supplementary Fig. S6). However, overexpression or knockdown of ZCCHC10 gene had no significant effects on cisplatin sensitivity in the p53-null (H358) or p53-mutant (H1437) cells (Fig. 5a). It has been reported that cisplatin-induced accumulation of p53 protein plays an important role in the response of lung cancer cells to cisplatin^21^. Therefore, we examined the effects of ZCCHC10 on p53 induction. The result showed that p53 protein levels dramatically augmented with the increase of concentration or time of cisplatin treatment in cells with endogenous or ectopically overexpressed ZCCHC10, but cisplatin-induced p53 protein significantly decreased in the cells lack of endogenous ZCCHC10 or ZCCHC10-suppressed cells (Fig. 5b, c). These results indicated that ZCCHC10 is required for p53 induction by cisplatin.

### ZCCHC10 interacts with and stabilizes p53

**Fig. 6:**
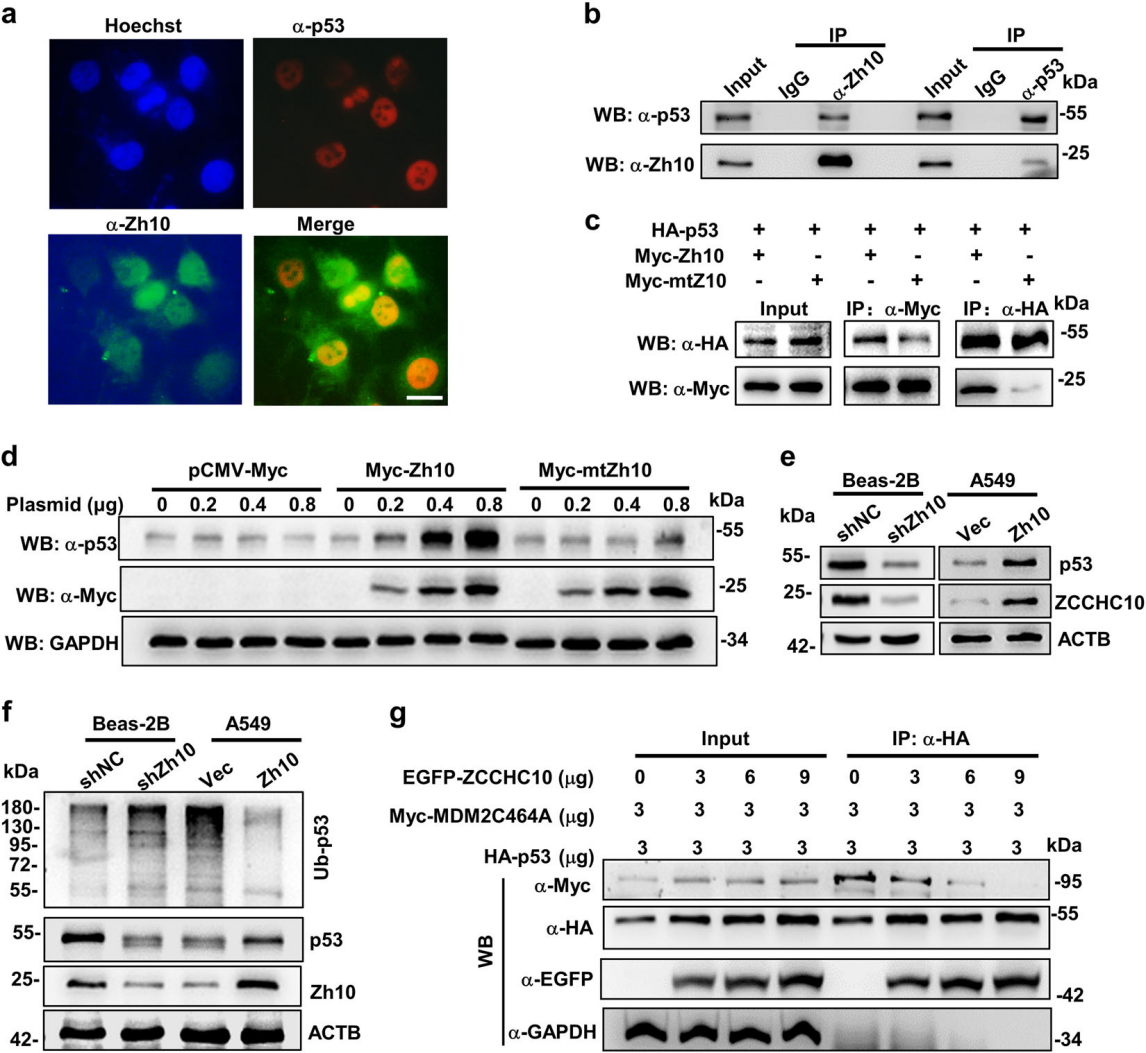
ZCCHC10 protein interacts and stabilizes p53 protein. **a** Immunofluorescence staining in Beas-2b cells. The murine anti-p53 monoclonal antibody and Texas Red-conjugated anti-mouse IgG (red) were used to detect p53, whereas rabbit anti-ZCCHC10 polyclonal antibody and FITC-conjugated anti-rabbit IgG (green) were used to detect ZCCHC10, respectively. Nuclei were stained by Hoechst 33258 (blue). Yellow in merged image represents colocalization of p53 and ZCCHC10 protein. Bar length: 10 μm. **b**, **c** Co-immunoprecipitation (co-IP) assays were performed in Beas-2b cells (**b**) or the Hek293 cells (**c**) co-transfected with HA-p53 and Myc-Zh10 (or Myc-mtZh10). Cell lysates were precipitated with the indicated antibody, and the immune complexes were subjected to WB. Input is equivalent to 10% of the lysate used for the co-IP. **d** WB analysis of p53 and Myc-ZCCHC10 protein in Hek293 cells at 24 h after transiently transfected with an increasing amount of empty vector, Myc-Zh10, or Myc-mtZh10 plasmids. **e** WB analysis of endogenous p53 and ZCCHC10 in Beas-2b stably expressing scramble shRNA (shNC) or ZCCHC10 shRNA (shZh10) and A549 cells stably expressing vector (Vec) or ZCCHC10 (Zh10). **f** Ubiquitination status of p53 following overexpression or knockdown of ZCCHC10. Cells were treated with MG132 for 4 h. After treatment, cell lysates (800 μg) were prepared and IPed with an anti-p53 antibody. Then, the precipitated proteins were subjected to WB using an anti-ubiquitin antibody to detect the ubiquitinated p53 (Ub-p53) protein (upper panel). Input is equivalent to 10% of the lysate used for the IP (lower panel). **g** Co-IP assays were performed in Hek293 cells co-transfected with the indicated amount of HA-p53, Myc-MDM2C464A, and EGFP-ZCCHC10 plasmids. At 24 h post transfection, cell lysates were prepared and IPed with rabbit anti-HA antibody, and the precipitated proteins were detected by WB analysis using a mouse anti-HA, anti-Myc, and anti-EGFP antibodies. Input is equivalent to 10% of the lysate used for the IP

ZCCHC10 has been shown to be a potential p53-interacting partner^17^. Therefore, we confirmed the interaction by immunofluorescence staining and co-IP assay. Immunofluorescence staining of Beas-2b showed that ZCCHC10 and p53 co-localized in the nuclei (Fig. 6a). Co-IP experiments were performed using the endogenous proteins in Beas-2b cells and the overexpressed proteins in Hek293 cells, respectively, both indicated the interaction of ZCCHC10 and p53 protein (Fig. 6b and Supplementary Fig. S7a).

CCHC-type zinc finger has been proved to participate in both DNA binding and protein–protein interactions^22^. Co-IP assay showed that mutation of the CCHC zinc finger in the ZCCHC10 protein reduced its interaction between with p53 (Fig. 6c), suggesting that the CCHC zinc finger is required for interaction of ZCCHC10 with p53.

Then, we investigated the influence of ZCCHC10 on p53 stability, and found that ZCCHC10 overexpression reduced the turnover of p53 protein (Supplementary Fig. S7b). Moreover, increasing wild-type ZCCHC10 expression significantly augmented p53 protein levels, but empty vector or CCHC-mutated ZCCHC10 rarely affected p53 protein levels (Fig. 6d), indicating that ZCCHC10-induces p53 stability depends on the interaction between p53 and ZCCHC10. We further detected effects of ZCCHC10 on endogenous p53 levels in lung cell lines. The results showed that overexpression of ZCCHC10 increased p53 level whereas knockdown of ZCCHC10 decreased p53 level (Fig. 6e and Supplementary Fig. S7c). However, knockdown or overexpression of ZCCHC10 did not affect the expression of mtp53 in p53-mutant H1437 cells (Supplementary Fig. S7d).

### ZCCHC10 suppresses the MDM2-mediated ubiquitination of p53 by disrupting the interaction between MDM2 and p53

The in vivo ubiquitination assay showed that the amount of ubiquitinated p53 in the A549 cells and H460 cells stably over-expressing ZCCHC10 were much lower than that in the corresponding control cells, whereas knockdown of ZCCHC10 promoted p53 ubiquitination in Beas-2B cells (Fig. 6f and Supplementary Fig. S7e). However, ubiquitination of mtp53 was not detected in H1437, also not affected by ZCCHC10 expression (Supplementary Fig. S7e). MDM2 is a p53-specific E3 ubiquitin ligase^3^. Hence, we performed a co-IP to detect the influence of ZCCHC10 on the interaction between p53 and MDM2. To eliminate the influence of MDM2-mediated p53 degradation on the interaction between p53 and MDM2, the ligase-inactive C464A mutant Myc-MDM2C464A^23^ was used in the co-IP experiment. As shown in Fig. 6g, the amount of p53-binding MDM2 decreased with the increase of ZCCHC10 expression, indicating that ZCCHC10 suppressed the interaction of p53 with MDM2. These results suggested that ZCCHC10 suppressed MDM2-mediated ubiquitination of p53 through disrupting the interaction between p53 and MDM2.

### ZCCHC10 plays roles through promoting the tumor-suppressive function of p53

**Fig. 7:**
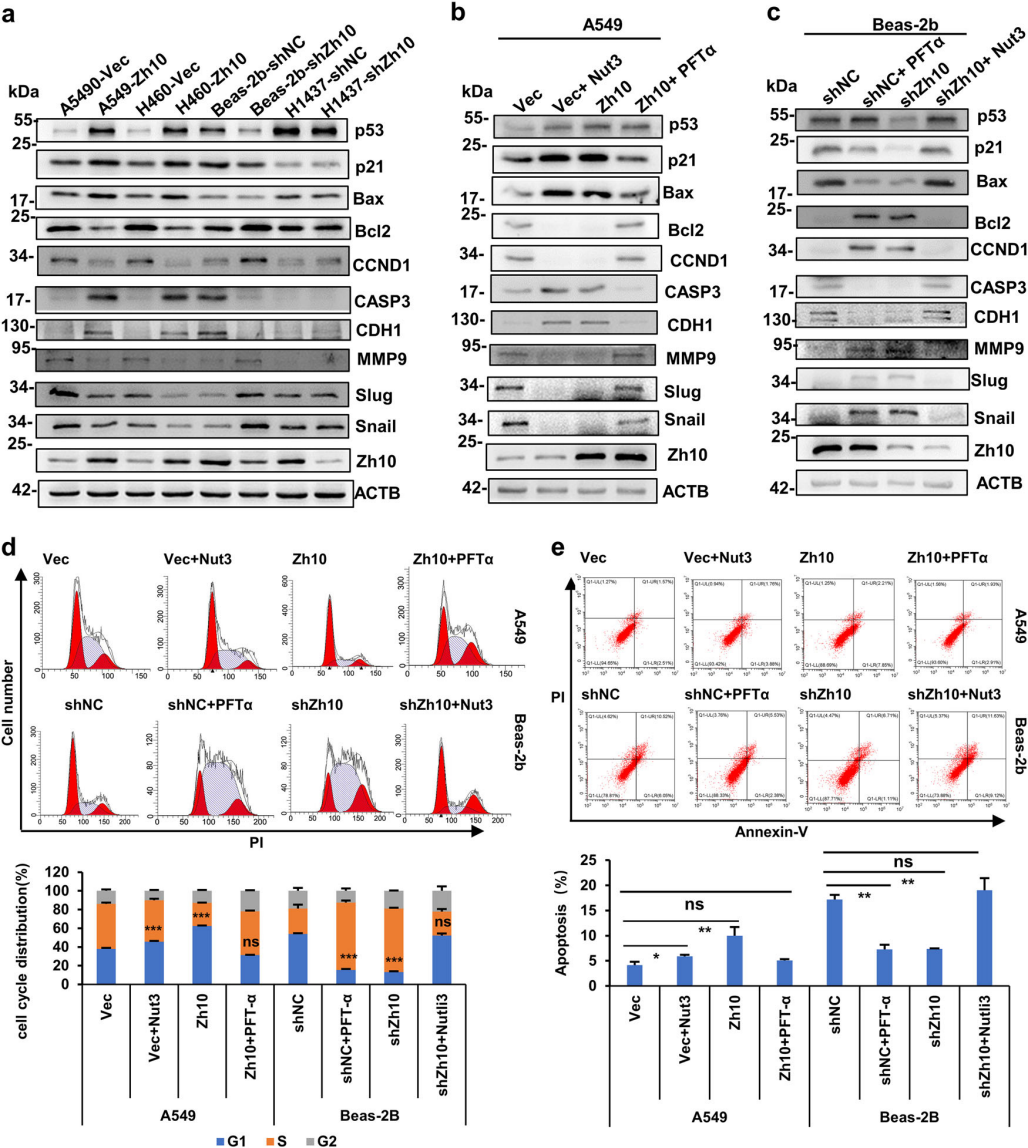
ZCCHC10 protein exerts tumor-suppressive function via p53. **a** WB analysis of p53, p21, Bax, Bcl2, CCND1, cleaved Caspase 3, CDH1, MMP9, Slug, and Snail proteins following overexpression or knockdown of ZCCHC10. **b**, **c** WB analysis of the indicated proteins after treatment with pifithrin-α (15 μM) or Nutlin3 (10 μM) for 72 h. **d**, **e** Flow cytometric analysis of cell cycle (**d**) and apoptosis (**e**) after treatment with pifithrin-α (15 μM) or Nutlin3 (10 μM) for 72 h. Representative images and corresponding statistical plots were shown in upper and lower panel, respectively. All the values were presented as means ± SD for three independent experiments

The p53 tumor suppressor mainly functions as a transcription factor^24^. Therefore, we investigated the influence of ZCCHC10 on transcriptional activity of p53. The luciferase reporter construct pp53-TA-luc, which contains a p53 response element, was used to monitor the transcriptional activity of p53. Based on the results of luciferase assay, overexpression of ZCCHC10 increased activity of p53 in cells with wtp53, but not in p53-null cells (Supplementary Fig. S8a, b). Moreover, mutation of CCHC domain of ZCCHC10 protein resulted in the decline of its influence on p53 activity (Supplementary Fig. S8c). Next, we detected the effects of ZCCHC10 on the mRNA and protein levels of the transcriptional targets (p21, Bax and Bcl2) of p53. Overexpression of ZCCHC10 significantly increased mRNA and protein levels of pro-apoptotic Bax and cell cycle inhibitor p21(CDKN1A) and decreased the expression of pro-survival Bcl2 in A549 cells; whereas ZCCHC10 knockdown exerted the opposite effects in Beas-2b cells, but did not have effects in p53-mutant H1437 cells (Fig. 7a and Supplementary Figs. S8d, e, f, and S9a). Additionally, ZCCHC10 enhanced the level of cleaved Caspase 3, but suppressed the expression of Cyclin D1 (CCND1) in the cells with wtp53 (Fig. 7a and Supplementary Fig. S9a). These results indicated that ZCCHC10 inhibited cell proliferation and survival through p53 pathway.

We have demonstrated that ZCCHC10 suppressed invasion and metastasis of lung cancer, whereas epithelial-mesenchymal transition (EMT) is a vital process in the invasion and metastasis of cancers. The p53 has been reported to directly or indirectly regulate the expression of various EMT markers, such as Snail (SNAI1)^25^, Slug (SNAI2)^26^, E-cadherin (CDH1)^27^. The results showed that ectopic expression of ZCCHC10 augmented the expression epithelial marker CDH1 and decreased the expression of mesenchymal markers (MMP9, SNAI1 and SNAI2). In contrast, knockdown of ZCCHC10 exhibited the opposite effects on these EMT markers. However, knockdown of ZCCHC10 had no significant effects on the EMT markers in p53-mutant cancer cells H1437 (Fig. 7a and Supplementary Fig. S9a).

Furthermore, the p53 inhibitor pifithrin-α attenuated the effects of ZCCHC10 overexpression on the expression of p53 and its targets, cell cycle, apoptosis and EMT, whereas the p53 activator Nutlin3 could reverse the effects of ZCCHC10 knockdown (Fig. 7b–e and Supplementary Fig. S9b, c).

The above results indicate that ZCCHC10 plays a tumor-suppressive role by activating p53 in lung cancers. Given that p53 gene is mutated in about half of lung cancers^28^, we analyzed ZCCHC10 expression and p53 mutation status in lung cancer tissues. Exons 5–9 of the p53 gene, the mutational hotspots^5^, were subjected to PCR amplification and sequencing. The expressions of ZCCHC10 and p21 were assessed by Western blotting. The results showed that ZCCHC10 protein levels in the cancer tissues with wtp53 were significantly lower than those in their corresponding adjacent noncancerous tissues, but there was no significant difference in its expression between the cancer tissues with mtp53 and their corresponding adjacent noncancerous tissues (Supplementary Fig. S10a, b). It suggested ZCCHC10 downregulation and p53 mutation were mutually exclusive. However, p21, a major indicator of p53 activity, was downregulated in all the lung cancer tissues, indicating that p53 inactivation occurs in the lung cancer tissues with mtp53, as well as in those with wtp53 (Supplementary Fig. S10a, c). Moreover, ZCCHC10 expression was positively correlated with p21 expression in the lung tissues with wtp53, but not in those with mtp53 (Supplementary Fig. S10d, e). Collectively, our data indicate that reduced ZCCHC10 expression contributes to inactivation of p53 pathway in lung cancer with wtp53.

## Discussion

In this study, we demonstrated that ZCCHC10 expression were statistically downregulated in LUAD tissues, and the patients with decreased ZCCHC10 mRNA predicted poorer survival. Functional studies showed that ZCCHC10 dramatically suppressed lung cancer cell proliferation, colony formation, migration, invasion, and cisplatin resistance in vitro, as well as tumor growth and metastasis in vivo. These results showed that ZCCHC10 is a novel tumor suppressor in lung cancer.

**Fig. 8:**
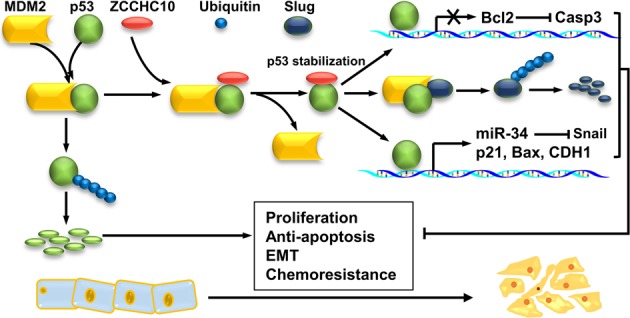
**A schematic diagram illustrating the tumor-suppressive mechanism of ZCCHC10**

However, the tumor-suppressive role of ZCCHC10 is dependent on the p53 status. Mechanistically, we demonstrated that ZCCHC10 stabilizes p53 protein by interfering with the MDM2-mediated ubiquitination of p53, and enhances the regulatory roles of p53 on the expression of the genes involved in cell cycle (p21), apoptosis (Bax, Bcl2) and EMT (E-cadherin, Snail and Slug). The p53 inhibitor pifithrin-α attenuates the influences of ZCCHC10 overexpression on p53 pathway, cell cycle, apoptosis, and EMT, whereas the p53 activator Nutlin3 can reverse the effects of ZCCHC10 knockdown. Based on our findings, ZCCHC10 probably is a co-activator of the tumor suppressor p53. The activation of p53 is essential for preventing abnormal cell proliferation and carcinogenesis^24^. Many tumor suppressors exert their roles by upregulating the stability or/and activity of the p53 protein, such as BAI1^8^ and LACTB^9^. Therefore, we proposed that ZCCHC10 play a tumor-suppressive role through stabilizing p53 protein: ZCCHC10 interacts with p53 and suppresses MDM2-mediated ubiquitination of p53 through disrupting the interaction between p53 and MDM2, leading to stability and accumulation of p53; once accumulated, p53 prevents oncogenic process by directly or indirectly regulating a series of genes involved in cell cycle (p21), apoptosis (Bax, Bcl2, Caspase 3) and EMT (CDH1, Snail, Slug) (Fig. 8).

Interestingly, ZCCHC10 expression is positively associated with survival of patients with LUAD, but not patients with LUSC. Moreover, ZCCHC10 expression has a greater benefit for non-smokers than smokers among patients with lung cancer. These differences in the prognostic effects of ZCCHC10 expression may be related to p53 status. TP53 mutation is more prevalent among patients with LUSC than patients with LUAD (57–65% versus 40–41%)^28,29^, and the frequency of TP53 mutation in the lungs of patients with cancer who smoke is also higher than in never smokers (48% versus 36%)^29^. Based on our results, ZCCHC10 functions in a p53-dependent manner, and ZCCHC10 expression is positively correlated with p53 activity in the lung tissues with wtp53, not in the tissues with mtp53. Therefore, ZCCHC10 expression has different prognostic impacts on patients with LUSCs and LUADs and between smokers and non-smokers.

In conclusion, we identified ZCCHC10 expression as a good prognostic factor for patients with lung cancer, particularly LUAD, and showed that ZCCHC10 exerts its tumor-suppressive effects by stabilizing the p53 protein in the lung cancer cells that harbor wtp53. Activation of p53 is an attractive strategy for anti-cancer therapy^30^. Thus, ZCCHC10 could be used a novel prognostic marker and therapeutic target in LUAD. To the best of our knowledge, this study is the first report describing the cellular function of the ZCCHC10 protein.

## Supplementary information

Supplementary tables and figures
