# Supplementary material for: ZCCHC10 suppresses lung cancer progression and cisplatin resistance by attenuating MDM2-mediated p53 ubiquitination and degradation
Source: Cell Death Dis. 2019 May 28;10(6):414. doi: 10.1038/s41419-019-1635-9 (PMC6538723; doi:10.1038/s41419-019-1635-9)
Supplement: Supplementary file 1 — Supplementary tables and figures [file 41419_2019_1635_MOESM1_ESM.pdf]

Supplementary tables and figures

Table S1. The clinicopathological parameters of the lung cancer patients used in the study

Gender	Age	Pathology	Differentiation	Diameter(cm)	TNM	Stage
Male	67	LUAD	moderate	2.5	T2N1M0	II
Male	69	LUAD	moderate	7	T3N2M0	IIIA
Female	45	LUAD	poor	2.5	T2N1M1	IV
Male	63	LUSC	poor	3	T2N0M0	I
Male	44	LUSC	moderate	1.5	T1N0M0	I
Male	58	LUSC	poor	1.5	T2N0M0	I
Female	55	LUAD	moderate	3	T1N1M0	II
Male	60	LUAD	moderate	3.5	T2N0M0	I
Male	66	LUAD	well	3	T1N1M0	II
Male	66	LUSC	poor	2.3	T1N0M0	I
Male	68	LUAD	poor	7	T3N0M0	II
Male	61	LUAD	poor	3	T1N0M0	I
Female	42	LUAD	moderate	4.5	T2N0M0	I
Male	44	LUAD	moderate	4.5	T2N1M0	II
Female	28	LUAD	moderate	3	T1N?M1	IV
Female	65	LUAD	moderate	2.5	T1N0M0	I
Female	63	LUAD	poor	3	T2N2M1	IV
Male	62	LUSC	poor	4	T2N2M0	III
Male	52	LUSC	poor	3	T1N1M0	II
Male	63	LUSC	poor	3.2	T2N0M0	I
Male	59	LUAD	poor	3	T2N0M0	I
Male	74	LUAD	moderate	2	T1N0M0	I
Male	59	LUAD	poor	3	T2N2M0	III
Female	60	LUAD	moderate	3	T2N2M0	III
Male	48	LUAD	poor	3	T1N0M0	I
Female	57	LUAD	moderate	2	T1N1M0	II
Male	57	LUSC	poor	6	T3N0M0	II
Female	52	LUAD	moderate	2	T1N2M0	III
Male	53	LUAD	moderate	2.2	T2N0M0	I
Male	51	LUAD	poor	5	T3N0M0	III
Male	73	LUAD	moderate	3	T1N0M0	I
Male	62	LUSC	moderate	4.5	T2bN0M0	IIA
Male	67	LUSC	moderate	1.5	T1bN0M0	IA2
Male	59	LUSC	moderate	6	T3N0M0	IIB
Male	70	LUSC	Moderate/ poor	6	T3N2M0	IIIB
Male	55	LUSC	Moderate/ poor	3.5	T2aN2M0	IIIA
Male	49	LUSC	Moderate/ poor	9	T4N0M0	IIIA
Male	71	LUSC	Moderate/ well	1.5	T1bN0M0	IA2
Male	64	LUSC	moderate	3	T1cN0M0	IA3
Male	61	LUSC	moderate	6	T3N0M0	IIB
Male	59	LUSC	moderate/ poor	4.5	T2bN0M0	IIA
Male	50	LUSC	moderate/ poor	3.5	T2aN0M0	IB
Female	59	LUAD	moderate	2.5	T2N0M0	IB
Female	45	LUAD	moderate	1.5	T1bN2M0	IIIA

Table S1 (continued). The clinicopathological parameters of the lung cancer patients used in the study

Gender	Age	Pathology	Differentiation	Diameter(cm)	TNM	Stage
Male	43	LUAD	moderate/ well	2.1	T1cN0M0	IA3
Female	65	LUAD	X	4	T2aN0M0	IB
Female	52	LUAD	moderate	2.4	T1cN0M0	IA3
Female	70	LUAD	moderate	1.8	T1bN2M0	IIIA
Female	46	LUAD	moderate	1.5	T1bN0M0	IA2
Male	67	LUAD	moderate/ poor	2	T1bN2M0	IIIA
Male	65	LUAD	moderate	1.5	T1bN0M0	IA2
Female	41	LUAD	moderate/ poor	5	T2bN2M0	IIIA
Female	74	LUAD	moderate/ well	2	T1bN0M0	IA2
Female	72	LUAD	moderate	2.5	T1cN0M0	IA3

Abbreviations: LUAD, adenocarcinoma; LUSC, squamous cell carcinoma.

Table S2. Antibodies and chemicals used in this study

**Name**	**Company**	**Catalog Number**
ZCCHC10	Sigma-Aldrich	HPA038944
p53	Abcam	Pab 1801
Bax	CST	2772
E-cadherin	CST	14472
Caspase 3	BBI	D120074
Myc-Tag rabbit antibody	CST	2278
Myc-tag mouse antibody	CMCTAG	AT0023
HA-Tag rabbit antibody	CST	3724
HA-tag mouse antibody	CMCTAG	AT0024
Texas Red-conjugated anti-mouse IgG	KPL	074-1806
FITC-conjugated anti-rabbit IgG	KPL	074-1506
Ubiquitin	Proteintech	10201-2-AP
ACTB	CMCTAG	AT0001
GFP	Abcam	AB6556
GAPDH	CMCTAG	AT0002
MMP9	CST	13667P
CyclinD1	Santa	J2411
Bcl-2	Wanleibio	wl01556
p21	ABclonal	A11877
SNAI2	ABclonal	A1057
SNAI1	ABclonal	A11794
Ki67	ABclonal	A11390
Nutlin-3	Apexbio	A4228
Pifithrin-α	Apexbio	A4206
Cisplatin	Selleck	S1166
MTT	Sangon	A100793

Table S3. The target sequences of ZCCHC10 shRNAs

**Name**	**Sequence (5′→3′)**
shZh-1/shZh10	GCTGAAGCAAATAAGCAACAT
shZh-2	GCAGTGGATTTCTAATGCTTT
shZh-3	GGTAACTGGTCATATATGACA
shZh-4	GGCTGCCTACAGAATATTATC

Table S4. Correlation of ZCCHC10 expression in tissue with patient's clinicopathological parameters

		Expression pattern		
Clinicopathological parameters		Normal expression (n=21)	Under-expression (n=33)	p-value [a]
Gender				0.0724
	Male	19	17	
	Female	2	16	
Age				0.8475
	<60y	12	15	
	>60y	9	18	
Subtype				0.0071**
	LUAD	6	29	
	LUSC	15	4	
Differentiation				0.6815
	Well/ Moderate	11	22	
	Poor	10	11	
Lymph node metastasis				0.6937
	N0	16	18	
	N1/N2	5	15	
Stage				0.3825
	I/II	18	20	
	III/IV	3	13	
Tumor size				0.0548
	≤3cm	9	24	
	>3cm	12	7	

a, Chi - square test (Fisher's Exact test); *, $p<0.05$

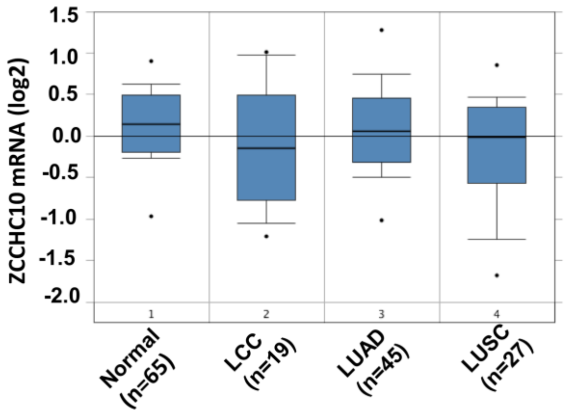

Fig. S1 Differential expression of ZCCHC10 mRNA between lung normal and tumor tissues were analyzed by Onocomine[1] based on the NCBI GEO dataset GSE19188[2]. LCLC: larger cell lung cancer; LUAD: lung adenocarcinoma; LUSC: lung squamous cell carcinoma.

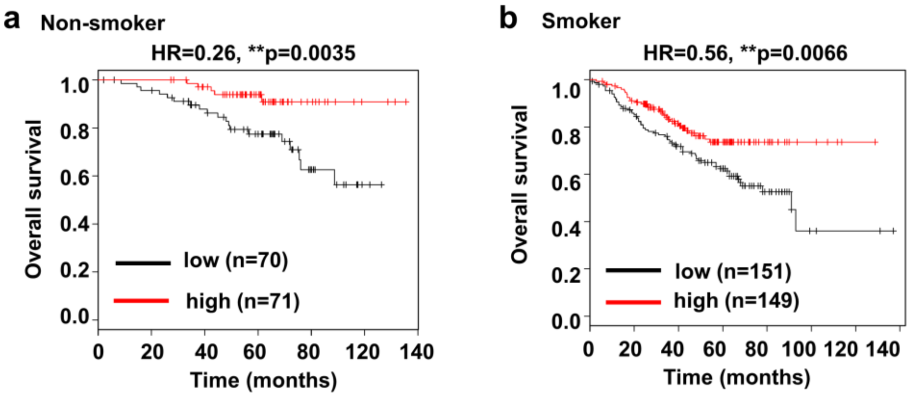

Fig. S2 Prognostic role of ZCCHC10 mRNA expression in lung cancer. Effects of ZCCHC10 mRNA expression in lung cancer tissue on patient survival was assessed using Kaplan Meier plotter based on the data from GEO, EGA and TCGA as described previously[3]. **a** The overall survival of the non-smoker patients with low or high ZCCHC10 mRNA expression. **b** The overall survival of the smoker patients with low or high ZCCHC10 mRNA expression. HR: hazard ratio; p: logrank p value.

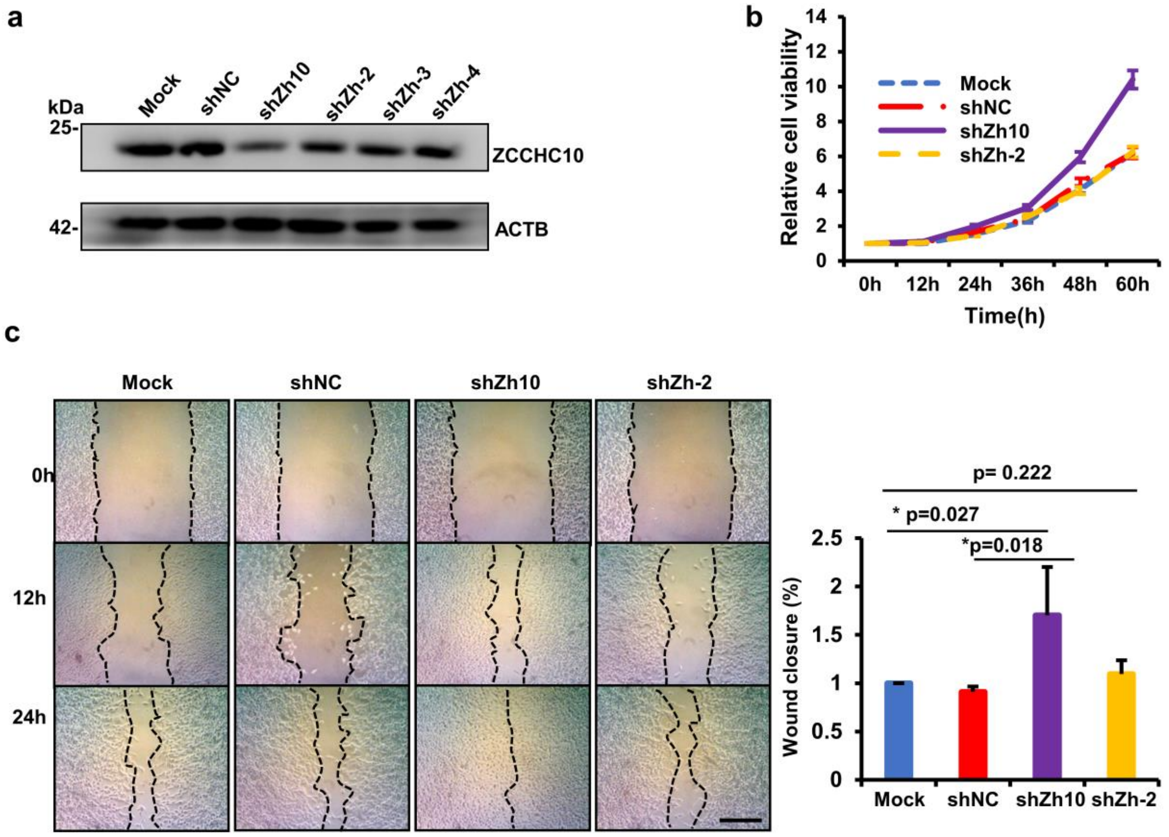

Fig. S3 Effects of ZCCHC10 interference on proliferation and migration of Hek293 cells. **a** ZCCHC protein levels in the untreated cells (Mock) and the cells stably expressing ZCCHC10 shRNAs (shZh-1, -2, -3 and -4) or scrambled shRNA (siNC). **b** The cell growth curves based on MTT assay. **c** Representative images (left panel) of wound area at indicated time and percentage of wound closure at 24 h after scratching (right panel). Bar length: 50 μm. Means ± SD shown are representative of two independent experiments and analyzed by two-tailed student t-test, *: $p \leq 0.05$; **: $p \leq 0.01$.

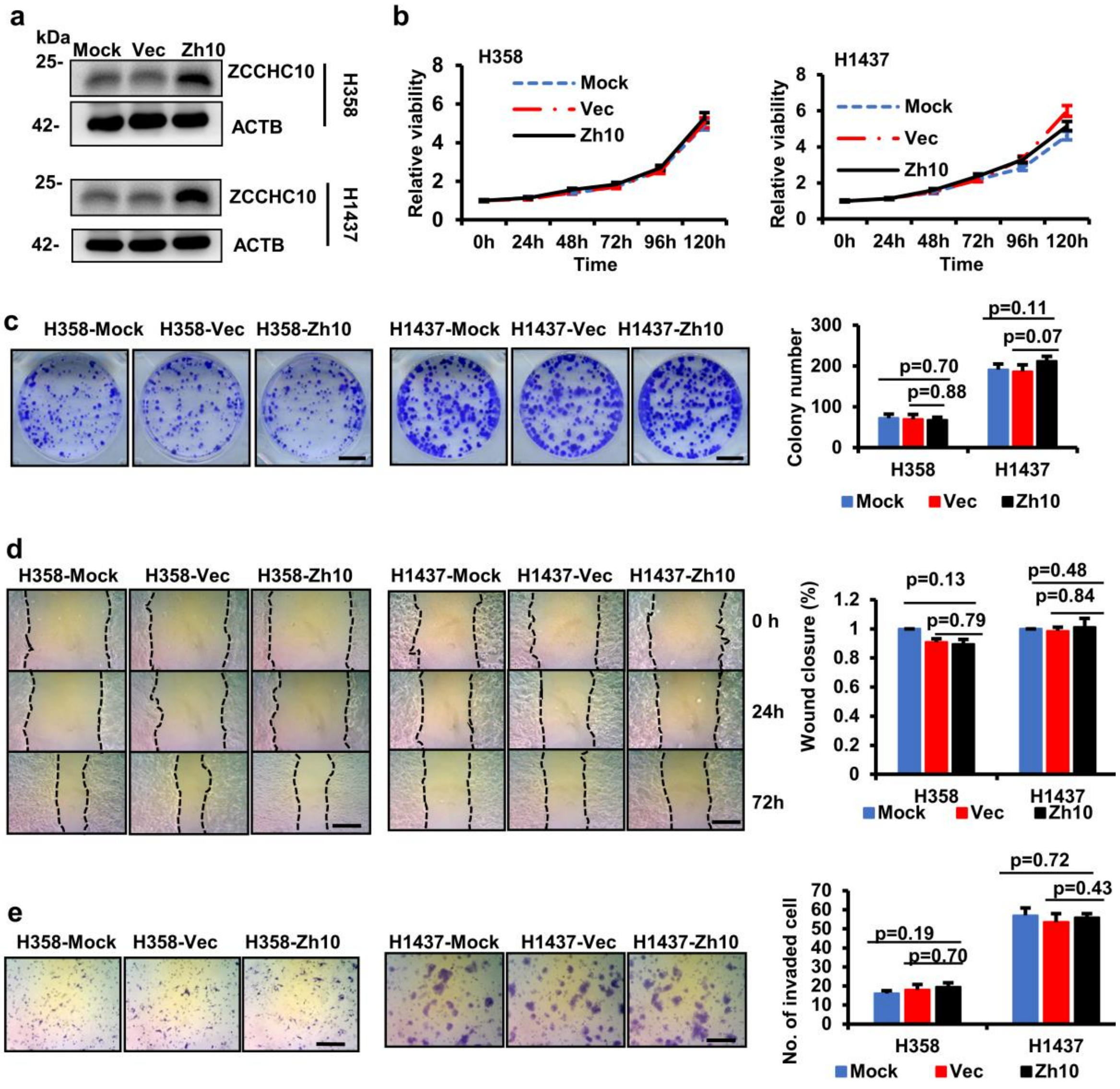

Fig. S4 Effects of ZCCHC10 overexpression on proliferation, migration and invasion of lung cancer cells H358 and H1437. **a** ZCCHC protein levels in the untreated cells (Mock) and the cells with stably expressing ZCCHC10 (Zh10) or empty vector (Vec). **b** The cell viability was determined by MTT assay. **c** Representative images of colony (left panel) and the colony numbers (right panel). Bar length: 1 cm **d** Representative images (left panel) of wound area at the indicated time and percentage of wound closure at 72h after scratching (right panel). Bar length: 50μm. **e** Representative images (left panel) and statistical analysis (right panel) of invaded cells in the Transwell invasion assay. Bar length: 50μm. All the values were presented as means ± SD for three independent in vitro experiments. Differences between two groups were analyzed by student t-test.

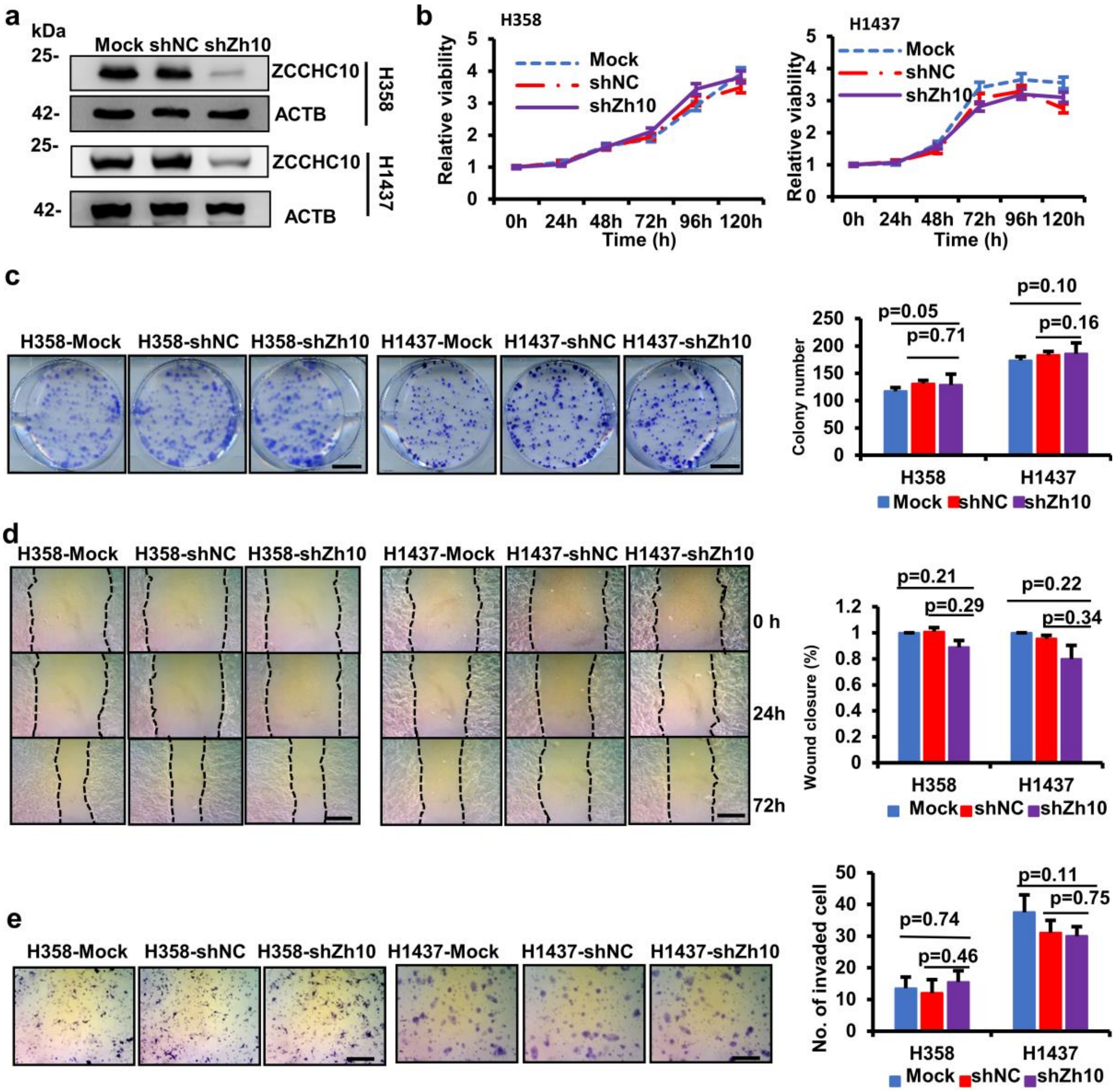

Fig. S5 Effects of ZCCHC10 knockdown on proliferation, migration and invasion of lung cancer cells H358 and H1437. **a** ZCCHC protein levels in the untreated cells (Mock) and the cells with stably expressing scrambled shRNA (siNC) or ZCCHC10 shRNA (shZh-1). **b** The cell viability was determined by MTT assay. **c** Representative images of colony (left panel) and the colony numbers (right panel). Bar length: 1 cm.**d** Representative images (left panel) of wound area at the indicated time and percentage of wound closure at 72h after scratching (right panel). Bar length: 50μm. **e** Representative images (left panel) and statistical analysis (right panel) of invaded cells in the Transwell invasion assay. Bar length: 50μm. All the values were presented as means ± SD for three independent in vitro experiments. Differences between two groups were analyzed by student t-test.

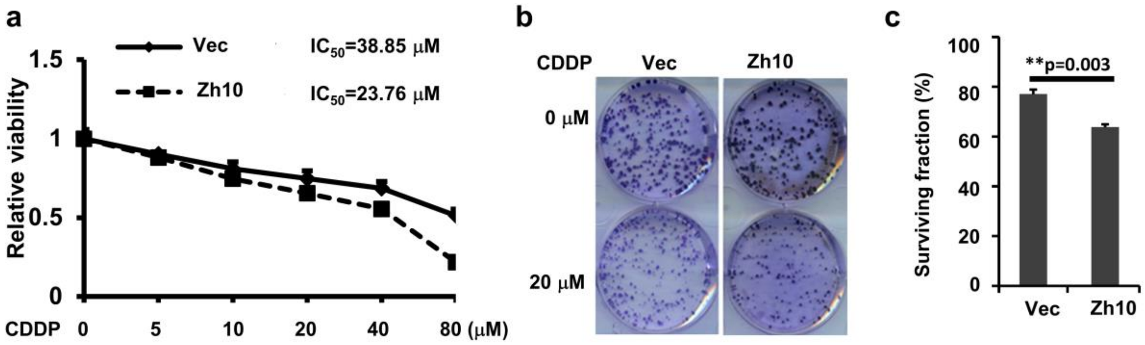

Fig. S6 Effects of ZCCHC10 knockdown on cisplatin sensitivity of HeLa cells. **a** The stable HeLa cells expressing ZCCHC10 (Zh10) or empty vector (Vec) were treated with different concentration of cisplatin for 72h. Cell viability was detected by MTT assay, IC50 values are representative of three independent in vitro experiments and calculated by SPSS statistics software. **b** Representative images of colony formation at different concentration of cisplatin. **c** Surviving fraction after cisplatin treatment based on colony formation assay.

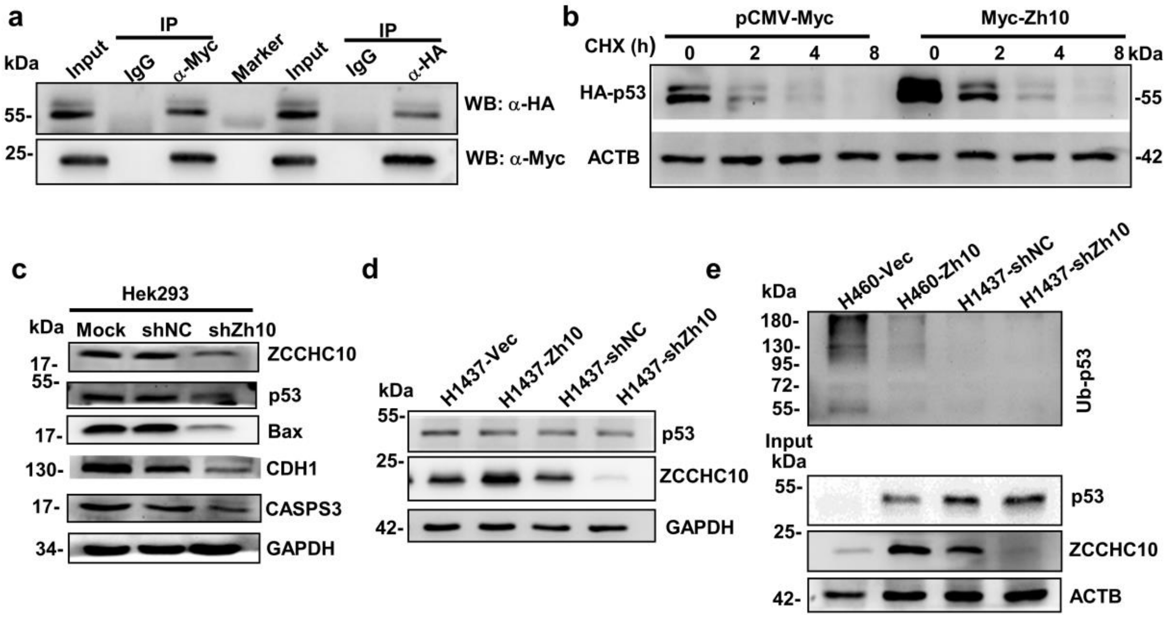

Fig. S7 ZCCHC10 protein interacts and stabilizes wildtype p53 protein. **a** Co-immunoprecipitation (IP) assay. Lysates were isolated from the HEK293 cells co-transfected with HA-p53 and Myc-ZCCHC10, and precipitated with control IgG, anti-Myc or anti-HA antibody. The immune complexes precipitated by anti-Myc (left panel) and anti-HA (right panel) antibodies were subjected to WB using anti-HA and

anti-Myc antibodies. **b** HA-p53 plasmids (0.2μg) were co-transfected with 0.8μg of empty vector pCMV-Myc or Myc-ZCCHC10. At 12 h post-transfection, transfected cells were incubated with cycloheximide (CHX, 50μg /ml) for indicated time, and then cell lysates were prepared and used for WB. **c** Influence of ZCCHC10 knockdown on the expressions of endogenous p53 and its downstream targets in HEK293 cells. **d** Influence of ZCCHC10 overexpression and knockdown on the expression of endogenous p53 in HEK293 cells. **e** Ubiquitination status of p53 in stable A549 cells and H1437 cells. Cells were treated with MG132 for 4 h. After treatment, cell lysates (800 μg) were prepared and immunoprecipitated (IPed) with an anti-p53 antibody. Then, the precipitated proteins were subjected to WB using an anti-ubiquitin antibody to detect the ubiquitinated p53 protein. Input is equivalent to 10% of the lysate used for the IP. siNC: cells stably expressing scrambled shRNA; shZh10: cells stably expressing ZCCHC10 shRNA-1; Vec: cells stably expressing empty vector; Zh10: cells stably expressing ZCCHC10.

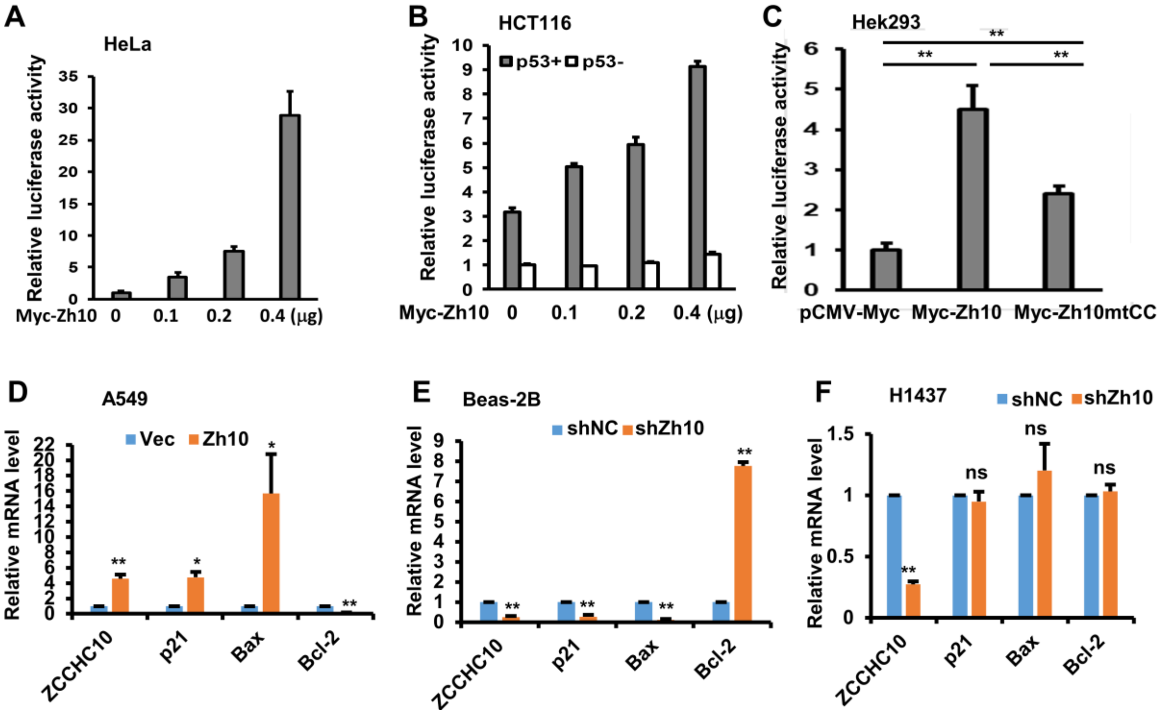

Fig. S8 ZCCHC10 protein stimulates the transcriptional ability of wildtype p53 protein. **a, b, c** Effects of ZCCHC10 expression on the activity of the p53-responsive luciferase reporter pp53-luc. Cells were seeded on 24-well plates, then a constant amount of pp53-Luc and pCMV-β were co-transfected with an increasing amounts of Myc-Zh10 (a, b) or 0.4 μg of the indicated plasmid (c). At 36h post-transfection, luciferase activities and β-galactosidase activities were measured, and luciferase activities were normalized to β-galactosidase activities. Data shown are means ± SD from four independent experiments. **d, e, f** Effects of ZCCHC10 expression on the mRNA levels of p53 downstream targets. RNA was extracted using Trizol reagent, and reverse-transcribed into cDNA. The SYBR green-based realtime PCR was performed using cDNA as template. The relative mRNA

expression levels were normalized to 18 S rRNA expression. Data shown are means ± SD from four independent in vitro experiments. Vec, the stable cells expressing empty vector; Zh10, the stable cells expressing ZCCHC10 gene; siNC, the stable cells expressing scrambled shRNA; shZh10, the stable cells expressing ZCCHC10 shRNA-1.

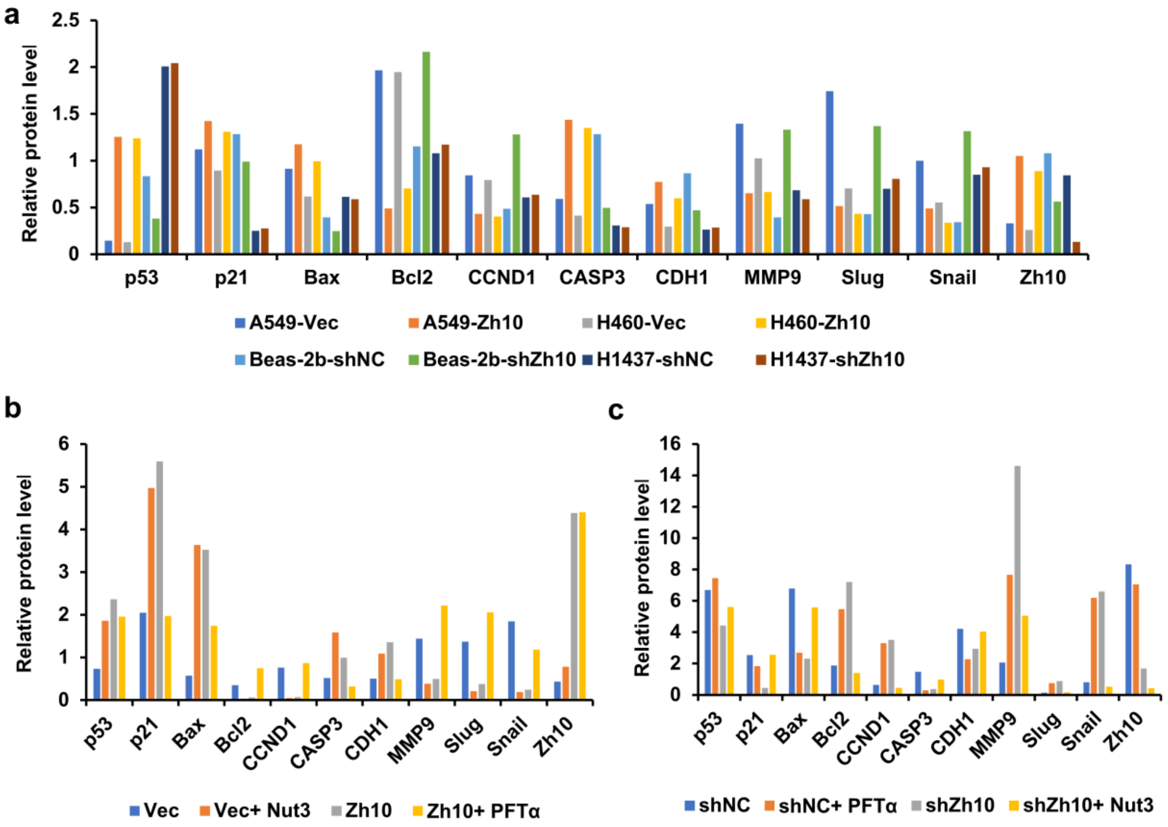

Fig. S9 Quantification of Western blots in Figure 7a, b, c. The band intensity in Western blots was determined by ImageJ. The values represent the relative protein levels normailzied to ACTB protein level. **a** Relative protein levels in Fig. 7a. **b** Relative protein levels in Fig. 7b. **c** Relative protein levels in Fig. 7c.

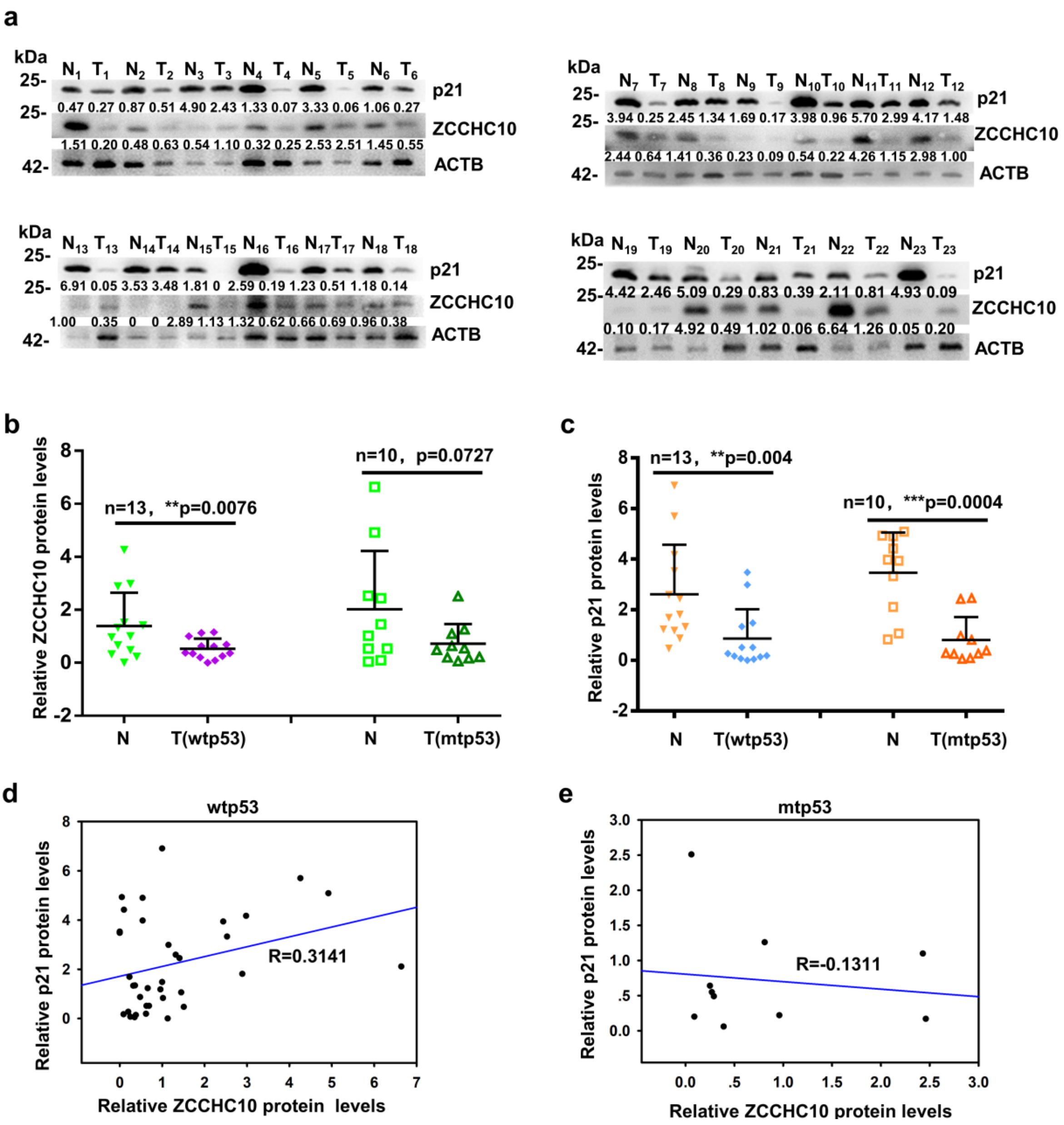

Fig. 10 Reduced expression of ZCCHC10 contributes the inactivation of p53 pathway in lung cancers. Exons 5-9 of the p53 gene, the mutational hotspots5, were subjected to PCR amplification and sequencing according to IARC protocol (http://p53.iarc.fr/ProtocolsAndTools.aspx). **a**.The expressions of ZCCHC10 and p21 in 23 pairs of lung cancer (T) and the matched noncancerous (N) tissues were assessed by Western blotting. The numbers below the blots indicate the relative levels of ZCCHC10 and p21 proteins normalized against that of ACTB. **b, c**. Differences in relative level of ZCCHC10 (**b**) or p21 protein (**c**) between lung cancer and the corresponding normal tissues were analyzed by GraphPad Prism software. **d, e**. Pearson's correlation coefficient (r) between p21 and ZCCHC10 in the lung tissue with wtp53 (**d**) or mtp53 (**e**).

References

1. Rhodes DR, Kalyana-Sundaram S, Mahavisno V, Varambally R, Yu J, Briggs BB, et al. Oncomine 3.0: genes, pathways, and networks in a collection of 18,000 cancer gene expression profiles. Neoplasia. 2007; 9: 166-80.
2. Hou J, Aerts J, den Hamer B, van Ijcken W, den Bakker M, Riegman P, et al. Gene expression-based classification of non-small cell lung carcinomas and survival prediction. PLoS One. 2010; 5: e10312.
3. Gyorffy B, Surowiak P, Budczies J, Lanczky A. Online survival analysis software to assess the prognostic value of biomarkers using transcriptomic data in non-small-cell lung cancer. PLoS One. 2013; 8: e82241.
